# The Antimicrobial Mechanism of Geraniol Against *Penicillium polonicum* and Its Application in Fresh-Cut Yam

**DOI:** 10.3390/antibiotics15050523

**Published:** 2026-05-21

**Authors:** Na Feng, Wei Yang, Xiaoyang Zhang, Yusha He, Min Zhang, Na Wang

**Affiliations:** College of Food Science and Bioengineering, Tianjin Agricultural University, Tianjin 300380, China; 15650226488@139.com (N.F.);

**Keywords:** geraniol, *Penicillium polonicum*, antifungal mechanism, fresh-cut yams, natural preservative

## Abstract

**Background:** Plant essential oils are extensively utilized for their antimicrobial properties; however, the specific antifungal mechanisms of certain compounds are not well characterized. Geraniol, a naturally occurring monoterpene alcohol approved for use in foods, demonstrates potential efficacy against spoilage fungi, yet detailed mechanistic insights are lacking. **Methods:** In this study, we determined the minimum inhibitory concentration (MIC) and minimum fungicidal concentration (MFC) of geraniol against *P. polonicum*. We assessed the underlying mechanisms by evaluating membrane integrity, intracellular leakage, reactive oxygen species (ROS), antioxidant enzymes (superoxide dismutase [SOD], peroxidase [POD], catalase [CAT]), malondialdehyde (MDA) levels, ATP content, and ATPase activity. Inoculated yam slices were exposed to geraniol vapor, and we monitored sensory, physicochemical, enzymatic, and microbial parameters. **Results:** Geraniol exhibited a minimum inhibitory concentration/minimum fungicidal concentration (MIC/MFC) of 0.3 mL/L. It disrupted cellular membranes, induced leakage, generated ROS, and caused lipid peroxidation, leading to elevated levels of malondialdehyde (MDA). Additionally, geraniol activated antioxidant enzymes and impaired energy metabolism. Fumigation with geraniol dose-dependently delayed the deterioration of yam, reduced weight loss, preserved texture and color, inhibited polyphenol oxidase (PPO) and POD activities, enhanced CAT and SOD activities, lowered MDA levels, and suppressed bacterial growth. **Conclusions:** Geraniol inhibits *P. polonicum* through multiple mechanisms, including membrane disruption, oxidative stress, and interference with energy metabolism, thereby effectively preserving the quality of fresh-cut yam and demonstrating potential as a natural preservative.

## 1. Introduction

*P. polonicum*, a member of the genus Penicillium, is prevalent in soil, air, and storage environments associated with various agricultural products. This fungus is a major pathogen responsible for the spoilage of fruits, grains, vegetables, and animal feed. It is a notable mycotoxin-producing organism, capable of synthesizing various mycotoxins, including the neurotoxic compound warfarin [1], which poses a considerable risk to food safety. Research indicates that the consumption of feed contaminated with this fungus can lead to kidney damage in laboratory animals [2]. In the food industry, *P. polonicum* is frequently found in products such as decaying yams [3], frozen chicken pieces [4], and applesauce [1], resulting in food spoilage and toxin accumulation that threaten human health.

Plant essential oils are volatile secondary metabolites derived from various plant tissues, including roots, leaves, flowers, fruits, seeds, and stems. These oils typically exist as oily liquids with distinctive aromatic scents, and their primary constituents comprise small-molecule compounds such as terpenes, phenols, aldehydes, and esters [5]. In comparison to conventional chemical preservation methods, plant essential oils are notable for their wide availability, safety, environmental friendliness, and broad antimicrobial spectrum, which has made them a focal point of current research in fields such as food preservation and medicine. Additionally, plant essential oils exhibit antifungal, anti-inflammatory [6], and antioxidant properties [7]. They are employed in the preservation of various meat products, including pork [8], ground meat [9], chicken [10,11], lamb [12], and other meats, as well as in the preservation of fruits such as papaya [13,14], strawberries [15], lychees [16], guavas [17], pineapples [18], and peaches [19]. Geraniol, a natural monoterpene compound, is widely present in the essential oils of various aromatic plants, including cardamom, rose, lemon, and geranium [20]. Geraniol is classified by the U.S. FDA (Food and Drug Administration) as Generally Recognized as Safe (GRAS) and is authorized as a food flavoring agent under China’s GB 2760-2024 [21]. The European Union has similarly approved its use in cosmetics and food in accordance with relevant regulations. Geraniol exhibits antimicrobial, anti-inflammatory, and antioxidant properties, and it also demonstrates antitumor, insecticidal, and antidepressant effects, along with certain neuroprotective benefits [22,23,24]. It can inhibit a range of pathogenic microorganisms, including Gram-positive bacteria such as *Streptococcus* spp. and *Staphylococcus aureus*, Gram-negative bacteria such as *Pseudomonas aeruginosa* and *Escherichia coli*, and fungi such as those causing *Citrus acid rot* and *Candida albicans* [25,26]. The minimum inhibitory concentration (MIC) and minimum fungicidal concentration (MFC) of Geraniol against the Citrus canker pathogen were both found to be 0.5 mg/mL. Following 12 h of Geraniol treatment, the spore germination rate decreased from 92.17% to 3.28%. Transmission electron microscopy revealed that Geraniol disrupted cell integrity, resulting in plasmolysis and vacuolization of organelles. Altered cell permeability led to the leakage of alkaline phosphatase and nucleic acids, as well as an increase in relative electrical conductivity. Furthermore, enhanced membrane lipid peroxidation and the accumulation of reactive oxygen species inhibited the pathogen’s ability to perform normal physiological activities [27]. Geraniol nanoemulsion (G-NE) effectively inhibits the growth of *Staphylococcus aureus* and *Escherichia coli* [28]. When Geraniol is combined with dodecanal, it induces wrinkling and deformation of the surfaces of Aspergillus spores and hyphae in pistachios. This interaction increases cell membrane permeability, resulting in the efflux of macromolecules, elevated extracellular conductivity, reduced ergosterol content in the membrane, and heightened intracellular levels of reactive oxygen species and malondialdehyde [29].

Fresh-cut yams are gaining popularity in the market due to their convenience and ready-to-eat characteristics. However, during processing and storage, they are highly susceptible to contamination by spoilage microorganisms. Among these, *P. polonicum* is a primary pathogen responsible for spoilage and deterioration [3]. While chemically synthesized preservatives are effective, they raise safety concerns. Consequently, this study investigates the antifungal mechanism of the natural plant essential oil component Geraniol against *P. polonicum* and explores its application in preserving freshly cut yam.

## 2. Results and Discussion

### 2.1. Determination of the MIC and MFC of Geraniol

**Figure 1:**
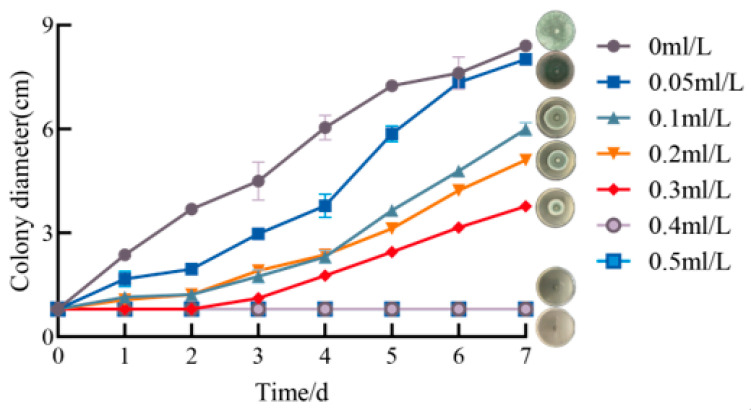
Inhibitory effect of Geraniol on bacterial colony growth.

Inhibitory effect of Geraniol on bacterial colony growth in Figure 1. After 48 h of Geraniol treatment, the colony diameter of *P. polonicum* in the control group measured 3.68 cm, while no mycelial growth was observed in the 0.3 mL/L Ger treatment group. This finding indicates that a concentration of 0.3 mL/L of Ger maintains effective antifungal activity for 48 h. Following 7 days of incubation, the colony diameter in the 0 mL/L group reached 8.4 cm, demonstrating rapid and uninhibited proliferation. In the 0.05 mL/L group, the colony diameter was 8.01 cm after 7 days; 0.1 mL/L group is 6 cm; 0.2 mL/L group is 5.1 cm; and in the 0.3 mL/L group, it is 3.77 cm. These results indicate that as the concentration of Geraniol increases, the inhibitory effect on *P. polonicum* growth strengthens progressively. The 0.4 mL/L and 0.5 mL/L concentrations exhibited complete inhibition, with no significant proliferation observed in the colony diameters of these treatment groups throughout the entire culture period. This suggests that this concentration range effectively blocks the growth of *P. polonicum*. Consequently, the minimum inhibitory concentration MIC and MFC of Ger against *P. polonicum* are 0.3 mL/L and 0.4 mL/L, respectively.

### 2.2. Effects of Geraniol on Mycelia and Spores

#### 2.2.1. Inhibitory Effect of Geraniol on the Growth of *P. polonicum* Mycelium

**Figure 2:**
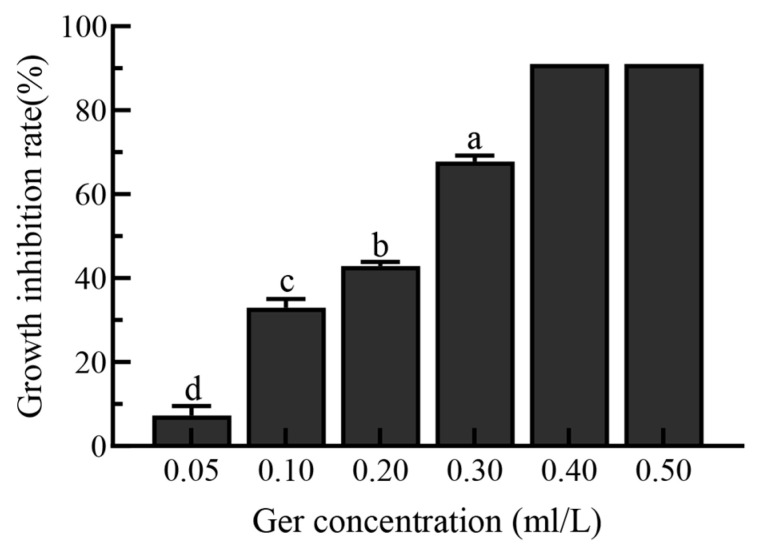
Inhibition rate of *P. polonicum* mycelial growth by Geraniol (*p* < 0.05). Different lowercase letters following the data indicate a statistically significant difference (*p* < 0.05).

The growth inhibition rate of Geraniol against *P. polonicum* demonstrated a concentration-dependent increase in Figure 2. In the low concentration range of 0.05–0.20 mL/L, the inhibition rate rose gradually with increasing concentration: it was 8% in the 0.05 mL/L group, increased to 32% in the 0.10 mL/L group, and reached 44% in the 0.20 mL/L group. At a concentration of 0.30 mL/L, the inhibition rate significantly increased to 68%. In the high-concentration range of 0.40–0.50 mL/L, the inhibition rate approached complete inhibition.

#### 2.2.2. Effects of Geraniol on the Morphology of *P. polonicum* Mycelia and Spores

**Figure 3:**
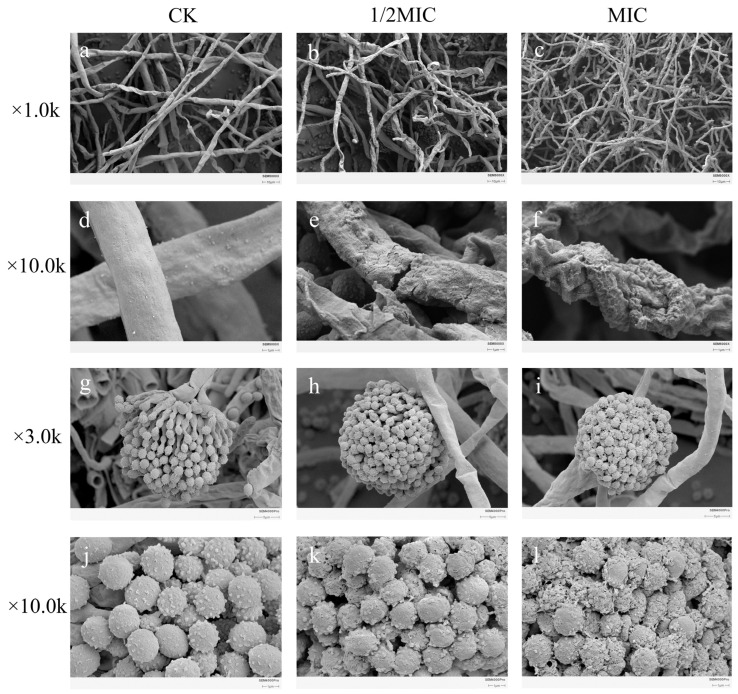
Effects of Geraniol on the morphology of *P. polonicum* hyphae and spores. Note: (**a**–**f**) Morphology of *P. polonicum* hyphaex; (**g**–**l**) *P. polonicum* conidiophore.

As the concentration of Geraniol increased, scanning electron microscope images revealed a progressively intensified destructive effect on the hyphae and spore structures of *P. polonicum* in Figure 3. Scanning electron microscopy revealed that the *P. polonicum* hyphae in the control group were smooth (Figure 3a), robust and extensively branched, whereas following treatment with geraniol at MIC (Figure 3c), the hyphal surface became markedly rough and shriveled, with the appearance of indentations and localized thickening. This indicates that the cell wall structure was disrupted, thereby inhibiting normal hyphal extension and colonization. Regarding spore morphology, spores in the control group (Figure 3d) were regular spheres with surfaces densely covered in distinct spiny projections; following treatment with the MIC (Figure 3f), the spores were severely shriveled and collapsed, with distorted and irregular shapes, and their characteristic spiny projections had almost completely disappeared. This disruption to spore structure directly affected spore viability and germination capacity. In summary, geraniol effectively inhibits fungal vegetative growth and the dissemination of propagules at the microscopic level by synergistically disrupting the integrity of the hyphal cell wall and the morphological structure of the spores. This provides direct morphological evidence for the potent antifungal activity observed at the macroscopic level.

#### 2.2.3. Effects of Geraniol on *P. polonicum* Cell Membranes

**Figure 4:**
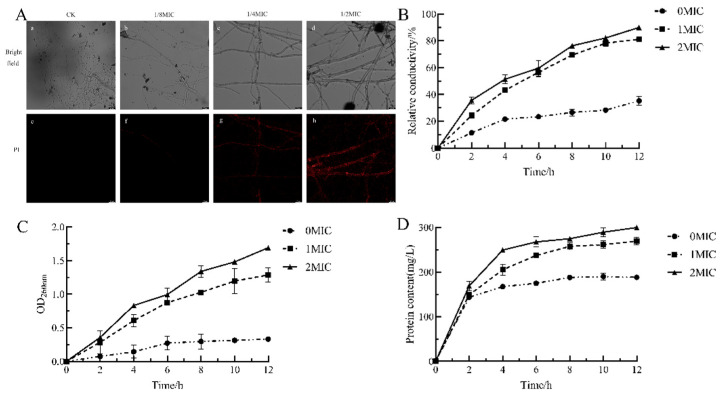
Effects of Geraniol on *P. polonicum* cell membranes. (**A**) Cell membrane integrity, (**B**) relative electrical conductivity, (**C**) nucleic acid leakage, and (**D**) protein leakage. Note: In Figure 4A, the red fluorescence is produced by the binding of PI dye to nucleic acids, indicating that the cell membrane has been disrupted. (**a**–**d**) show bright-field images, whilst (**e**–**h**) show the hyphae of Penicillium polonicum following PI staining.

Propidium iodide (PI) serves as a valuable tool for evaluating the integrity of pathogen cell membranes following drug treatment. When pathogen cell membranes are compromised, the PI dye can penetrate the hyphae and bind to nucleic acids, resulting in red fluorescence [30,31]. As shown in Figure 4A, fluorescence microscopy demonstrated that the hyphae in the control group (CK) remained morphologically intact and displayed no significant red fluorescence. In the 1/8 MIC treatment group, localized and faint fluorescence was observed; the 1/4 MIC treatment group exhibited increased fluorescence, with distinct hyphal outlines indicating considerable damage to the cell membrane under these conditions. The 1/2 MIC treatment group revealed the most intense fluorescence, with hyphal structures entirely disrupted and strong red signals evident within the hyphae. These findings suggest that Geraniol induces cell death by compromising cell integrity, enhancing cell membrane permeability, and facilitating the diffusion of the PI dye into the cells; moreover, this detrimental effect is concentration-dependent.

The cell membrane of pathogenic fungi functions as a semipermeable protective barrier. Exposure to external environmental stimuli or antimicrobial agents compromises its structure, resulting in increased cell permeability. Consequently, ions are released, which elevates the conductivity of the culture medium [32]. An increase in relative conductivity signifies that the permeability of the hyphal cell membrane has been disrupted [33]. In Figure 4B, the relative conductivity of all Geraniol treatment groups at varying concentrations exhibited an upward trend with prolonged treatment duration, although the rate of increase differed among groups. The 0 MIC group demonstrated the lowest relative conductivity, which increased gradually, reaching only 35.21% by 12 h. In contrast, the relative conductance of both the MIC and 2 MIC groups surpassed that of the control group and increased with higher Geraniol concentrations; the 2 MIC group displayed the highest relative conductance at all time points, achieving 89.95% at 12 h. These results indicate that Geraniol significantly enhances the permeability of *P. polonicum* cell membranes, and this effect shows a clear dose-dependent relationship.

The leakage of macromolecules, including proteins and nucleic acids, from cells serves as a critical indicator of cell membrane damage [34]. As illustrated in Figure 4C, the optical density (OD) at 260 nm for nucleic acid leakage in the 0 MIC (control) group remained the lowest throughout the experiment, reaching only 0.33 at 12 h, with a gradual increase observed over time. In contrast, the OD_260nm_ values in the MIC and 2 MIC groups exhibited significant increases as time progressed, with the 2 MIC group consistently displaying higher values than the MIC group at all time points. At 12 h, the OD_260nm_ value for the 2 MIC group was 1.69. These findings suggest that higher concentrations of Geraniol result in increased nucleic acid leakage.

Figure 4D presents data on protein leakage, where the protein content in the 0 MIC group increased relatively slowly, reaching 188.16 mg/L at 12 h. Conversely, in the MIC and 2 MIC groups, protein content initially rose rapidly before stabilizing, with the 2 MIC group consistently exhibiting higher values than the MIC group at all time points. At 12 h, the protein content in the 2 MIC group was 299.6 mg/L, compared to 269.25 mg/L in the MIC group. In summary, Geraniol can induce the leakage of nucleic acids and proteins in *P. polonicum*. Damage to the cell membrane was more pronounced with high-concentration treatment, suggesting that Geraniol may exert its antifungal effect by compromising cell membrane integrity, which leads to the release of intracellular substances, including nucleic acids and proteins.

#### 2.2.4. Effects of Geraniol on the Reactive Oxygen Species Metabolism of *P. polonicum*

**Figure 5:**
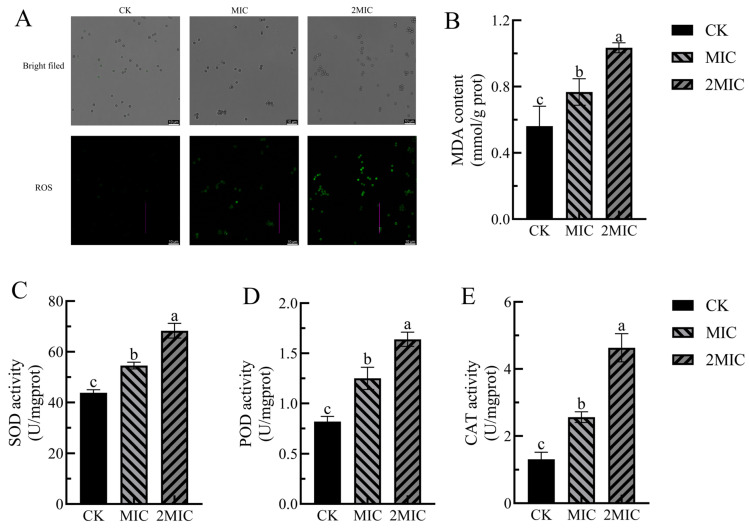
Effects of Geraniol on reactive oxygen species metabolism in *P. polonicum*. (**A**) Reactive oxygen species levels, (**B**) MDA content, (**C**) SOD activity, (**D**) POD activity, and (**E**) CAT activity. Different lowercase letters following the data indicate a statistically significant difference (*p* < 0.05).

The 2′,7′-dichlorofluorescein diacetate (DCHF-DA) dye is a widely utilized indicator for assessing intracellular reactive oxygen species (ROS) levels. This dye can penetrate the cell membrane and enter the cytoplasm, where it is hydrolyzed by intracellular esterases to yield 2′,7′-dichlorofluorescein dihydrogen (DCFH). Subsequently, DCFH is rapidly oxidized by intracellular ROS to produce 2′,7′-dichlorofluorescein (DCF), a compound characterized by increased fluorescence intensity, which is employed to detect ROS accumulation within cells [35]. As illustrated in Figure 5A, bright-field microscopy revealed no significant differences in bacterial morphological characteristics among the CK group, MIC group, and 2 MIC group. However, ROS fluorescence imaging demonstrated that the CK group exhibited very few green fluorescent spots, while the MIC group displayed a marked increase in the number of fluorescent spots. The 2 MIC group showed an even greater increase in both the number and brightness of fluorescent spots. These findings suggest that Geraniol induces ROS accumulation in *P. polonicum* in a dose-dependent manner, with more pronounced oxidative stress observed under the 2 MIC treatment. It is posited that the ROS burst represents a critical intermediate step in Geraniol’s disruption of the cell membrane and the subsequent leakage of intracellular substances, thereby providing evidence for this mechanism at the level of oxidative stress.

MDA serves as a crucial marker for evaluating oxidative stress levels, with its concentration serving as an indirect indicator of oxidative damage to cell membranes [36]. In Figure 5B, the MDA level in the control group was measured at 0.56 mmol/prot. In contrast, following treatment with Ger at concentrations of MIC and 2 MIC, MDA levels rose to 0.77 and 1.03 mmol/prot, respectively. These findings suggest that Geraniol treatment intensified the ROS burst in *P. polonicum*, increased lipid peroxidation, enhanced MDA production, and demonstrated a dose-dependent effect.

Living organisms possess a comprehensive antioxidant defense system that includes various enzymes, such as POD, SOD, and CAT. These enzymes scavenge reactive oxygen species through catalytic action, thereby protecting cells from oxidative damage [37]. Figure 5C–E illustrates that, compared to the control group, the activities of all three antioxidant enzymes exhibited an upward trend following treatment with both MIC and 2 MIC of Geraniol, with values significantly higher than those of the control group (*p* < 0.05). Specifically, after treatment with the 2 MIC of Geraniol, SOD activity increased by 55.86%, POD activity rose by 100%, and CAT activity surged by 253.44% compared to the control group. These results indicate that Geraniol treatment significantly enhances the activity of SOD, POD, and CAT enzymes within the antioxidant defense system of *P. polonicum*.

#### 2.2.5. Effects of Geraniol on the Mitochondria of *P. polonicum*

**Figure 6:**
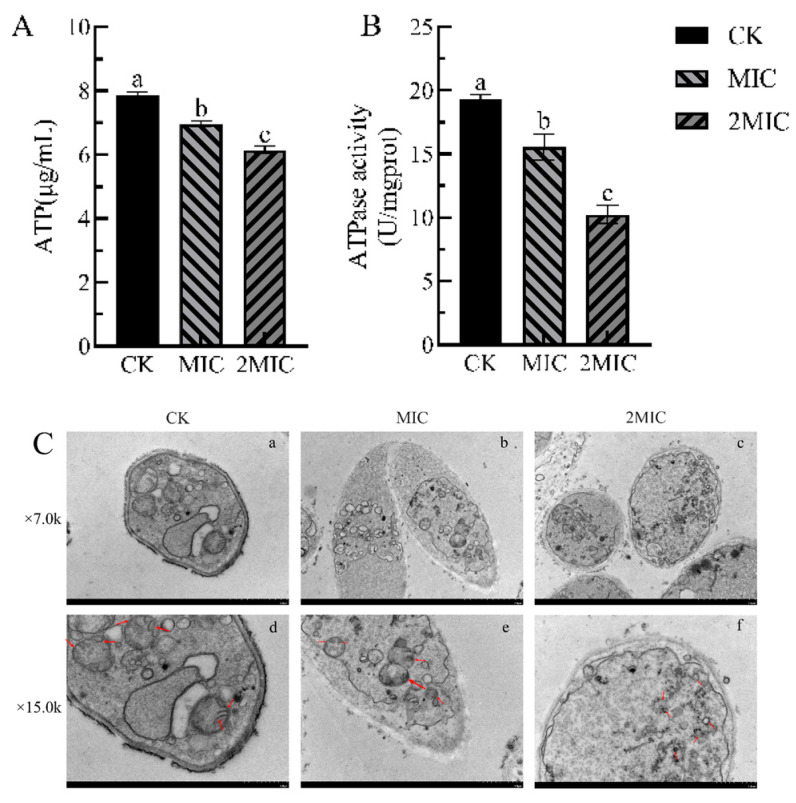
Effects of Geraniol on the mitochondria of *P. polonicum*. (**A**) ATP content, (**B**) ATPase activity, (**C**) mitochondrial ultrastructure. Note: In Figure 6C, (**a**–**f**) indicate intact or ruptured mitochondria and cell membrane. Different lowercase letters following the data indicate a statistically significant difference (*p* < 0.05).

Changes in intracellular and extracellular ATP levels can indicate irreversible damage and disruption to the mitochondrial membrane [38]. Geraniol treatment significantly impairs the energy metabolism system of *P. polonicum*, with the inhibitory effect intensifying at higher concentrations (Figure 6A). The control group exhibited the highest ATP content at 7.85 μg/mL; however, following treatment with the minimum inhibitory concentration (MIC) and 2 MIC of Geraniol, ATP levels decreased to 6.96 μg/mL and 6.14 μg/mL, respectively, both of which were significantly lower than those of the control group (*p* < 0.05). These findings suggest that Geraniol induces an insufficient cellular energy supply in the fungus by inhibiting ATP synthesis or accelerating its degradation.

ATPase activity serves as a reflection of the pathogen’s energy supply and overall metabolic status Figure 6B. ATPase activity in the control group was measured at 19.33 U/mg prot; after treatment with MIC and 2 MIC of Geraniol, enzyme activity declined to 15.54 U/mg prot and 10.24 U/mg prot, respectively. These results indicate that Geraniol directly inhibits ATPase activity, thereby disrupting energy conversion processes. In summary, Geraniol disrupts the normal energy metabolic balance of *P. polonicum* by reducing ATP levels and inhibiting ATPase activity, ultimately impairing the bacterial energy supply and inhibiting growth and reproduction.

Mitochondria serve as the central organelles in cellular energy metabolism, and the integrity of their structure directly influences the growth and reproduction of pathogens [39]. As illustrated in Figure 6C, transmission electron microscopy (TEM) observations indicated that the mitochondria of *P. polonicum* in the control group displayed clear and intact structures, characterized by distinct double membranes, well-organized cristae, and a uniform, dense matrix. In contrast, mitochondria of *P. polonicum* treated with the minimum inhibitory concentration (MIC) of Geraniol (Figure 6C(e)) exhibited swelling, with some cristae appearing blurred and a reduction in matrix density. Following treatment with 2 MIC of Geraniol, the overall cellular structure was severely compromised: the double-membrane architecture was nearly completely disintegrated, the cristae were absent, the matrix leaked, and extensive vacuolated regions emerged (Figure 6C(f)). These findings demonstrate that Geraniol can inhibit the growth and reproduction of the fungus by disrupting mitochondrial structure, thereby impairing or abolishing its energy metabolism function.

### 2.3. The Effect of Geraniol on the Quality of Fresh-Cut Chinese Yams

#### 2.3.1. Effect of Geraniol on the Sensory Evaluation of Fresh-Cut Chinese Yams

**Figure 7:**
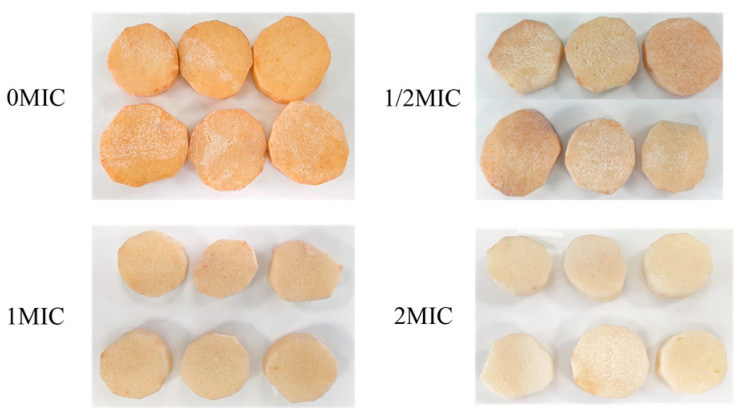
Fresh-cut yam stored for 15 days.

Sensory evaluation scores exhibited a general decline (Table 1). The control group, designated as the 0 MIC group, experienced the most pronounced decrease in sensory scores, which fell to 78.25 after 3 days of storage and further declined to 45.65 after 15 days. In contrast, the groups treated with Geraniol (1/2 MIC, MIC, 2 MIC) demonstrated a considerably smaller reduction in sensory scores compared to the control group. Notably, as the concentration of Geraniol increased, the sensory scores improved. After 15 days of storage, the 1/2 MIC group recorded a score of 60.34, the MIC group scored 70.12, and the 2 MIC group achieved a score of 80.35. As illustrated in Figure 7, the fresh-cut yam in the 0 MIC group displayed significant browning and a softened texture after 15 days of storage, while the fresh-cut yams in the 2 MIC group maintained the best color, appeared the freshest, and exhibited superior texture. In conclusion, Geraniol effectively preserves the sensory quality of fresh-cut yam; concentrations up to 2 MIC correlate with enhanced sensory quality.

#### 2.3.2. Effect of Geraniol on the Appearance Quality of Fresh-Cut Yams

**Figure 8:**
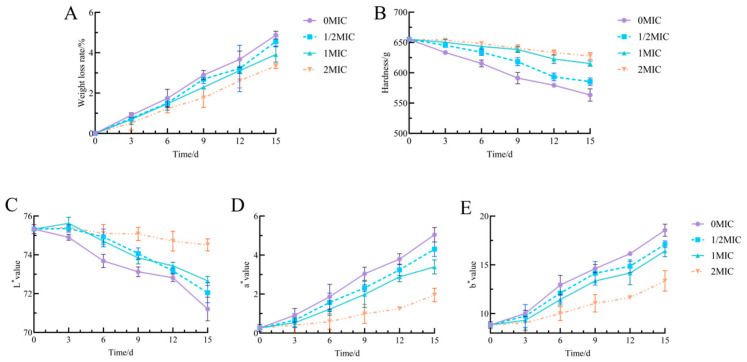
Effect of Geraniol on the quality of fresh-cut yam. (**A**) weight loss, (**B**) firmness, and (**C**–**E**) color difference.

As storage time increased, the rate of weight loss in fresh-cut yam samples from each treatment group gradually escalated (Figure 8A). The 0 MIC group exhibited the most significant increase in weight loss, reaching 4.87% after 15 days. In contrast, the weight loss rates in all treatment groups supplemented with Geraniol (1/2 MIC, MIC, 2 MIC) were lower than those in the control group. Moreover, within the 2 MIC range, a higher concentration of Geraniol corresponded to a more pronounced inhibitory effect on weight loss; at 15 days, the weight loss rates for the MIC and 2 MIC groups were 3.91% and 3.34%, respectively. These results suggest that Geraniol treatment effectively mitigates water loss in fresh-cut yams, thereby influencing quality changes during storage.

As storage time increased, the firmness of fresh-cut yams in each group decreased; however, the impact of treatment with varying concentrations of Geraniol on firmness exhibited notable differences (Figure 8B). The 0 μM group experienced the most significant reduction in firmness, decreasing from an initial 655.33 g to 563.48 g after 15 days. In contrast, treatments with MIC and 2 MIC Geraniol showed no significant difference in hardness after 9 days of storage, with a mere variation of 2.18 g. The 2 MIC treatment group consistently demonstrated the highest firmness, measuring 627.81 g after 15 days of storage. The MIC treatment group followed with a firmness of 615.27 g, while the 1/2 MIC treatment group recorded 585.48 g, which was 22 g greater than the control group. These results suggest that Geraniol can effectively reduce the decline in firmness of fresh-cut Chinese yam, with the protective effect becoming more pronounced at higher concentrations.

As storage time increased, the L* values of fresh-cut yams in each treatment group exhibited a downward trend, indicating a gradual decrease in brightness (Figure 8C). Notably, the L* value of the control group decreased most significantly, reaching 71.21 by day 15. In contrast, the decline in L* values for the 1/2 MIC, MIC, and 2 MIC treatment groups was less pronounced, revealing a concentration-dependent pattern; higher concentrations of Geraniol resulted in better maintenance of L* values. By day 15 of storage, the L* values for the MIC and 2 MIC treatment groups were 72.66 and 74.51, respectively. The a* values for all treatment groups exhibited an overall upward trend during storage, indicating a shift in color towards red tones, which resulted in browning (Figure 8D). The a* value of the control group increased most rapidly, indicating that the fresh-cut Chinese yam in this group reddened more quickly, reaching an a* value of 5.04 by day 15. However, the increase in a* values for the Geraniol-treated fresh-cut yam samples was significantly smaller than that of the control group, and the rate of increase in a* values diminished as the concentration increased. As storage time increased, the b* values for all treatment groups exhibited an upward trend, signifying that the fresh-cut yam samples were transitioning toward a yellow hue (Figure 8E). The most significant increase in b* value occurred in the control group, which rose from an initial value of 8.84 to 18.55 after 15 days. In contrast, the 1/2 MIC, MIC, and 2 MIC treatment groups demonstrated a more gradual increase, reaching values of 8.27, 7.58, and 4.52, respectively, after 15 days of storage. The combined analysis of the L*, a*, and b* values suggests that Geraniol effectively controls color changes in fresh-cut Chinese yam, thereby slowing the decline in brightness and inhibiting both reddening and yellowing.

#### 2.3.3. Determination of the Effect of Geraniol on the Enzymatic Activity of Fresh-Cut Yam

**Figure 9:**
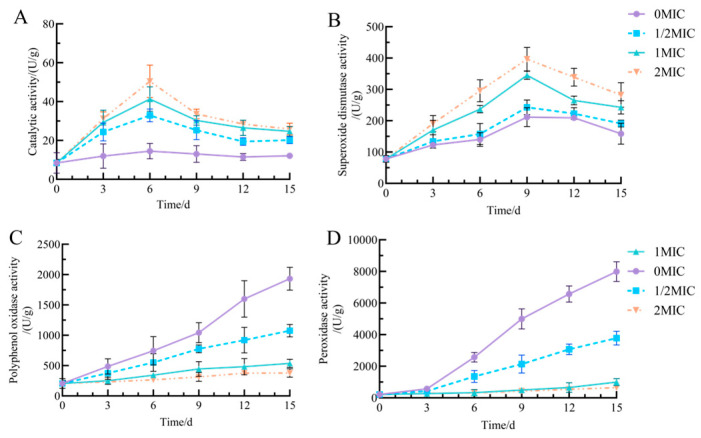
Effect of Geraniol on enzyme activity in fresh-cut yam. (**A**) CAT, (**B**) SOD, (**C**) PPO, and (**D**) POD.

Catalase (CAT) is a vital enzyme that significantly contributes to the antioxidant defense system of fresh-cut Chinese yam, with variations in its activity indicating the yam’s capacity to scavenge hydrogen peroxide [40]. As storage time increases, CAT activity in all groups of fresh-cut yam initially rises before subsequently declining (Figure 9A). After 6 days of storage, the 2 MIC group exhibited the most pronounced changes in CAT activity, achieving a peak of 50.64 U/g during the early phase. Following 15 days of storage, this group experienced the smallest decline, stabilizing at 25.87 U/g. In contrast, the 0 μM group demonstrated a relatively modest initial increase, reaching 14.53 U/g at 6 days, followed by a decrease to 12.13 U/g at 15 days. Geraniol significantly induced CAT activity (*p* < 0.05), which is crucial for enhancing the intrinsic antioxidant capacity of fresh-cut yam and alleviating oxidative damage.

SOD plays a vital role in scavenging superoxide anion radicals, with changes in its activity serving as a direct indicator of the body’s antioxidant status [41]. As storage time increased, the SOD activity of fresh-cut yam across all groups demonstrated an initial rise followed by a subsequent decline (Figure 9B). The 2 MIC group exhibited the most significant increase in SOD activity during the early phase, reaching 396.43 U/g by day 9, and the smallest decline in the later phase, stabilizing at 282.39 U/g by day 15. The trends in SOD activity for the 1/2 MIC and MIC groups fell between those observed in the 2 MIC and 0 MIC groups. The 0 MIC group displayed a relatively modest increase in the early stage, achieving only 211.39 U/g at day 9, followed by a pronounced decline to 158.55 U/g by day 15. In summary, Geraniol effectively regulates the synthesis and degradation of SOD in fresh-cut yam, thereby enhancing the stability of SOD activity and preventing the excessive accumulation of harmful substances, such as reactive oxygen species, within the tissue.

The results of the PPO activity assay are presented in Figure 9C. As storage time increased, the PPO activity of fresh-cut yams in each group exhibited an upward trend. The 0 MIC group demonstrated the most pronounced increase in PPO activity, reaching 1931.67 U/g after 15 days. In contrast, the increases in PPO activity for the treatment groups supplemented with 1/2 MIC, MIC, and 2 MIC of Geraniol were significantly lower than that of the control group (*p* < 0.05). Specifically, after 15 days, the PPO activity in the MIC group was measured at 536.67 U/g, while the 2 MIC group recorded 378.33 U/g. These results indicate that Geraniol effectively delays the browning of fresh-cut yam by inhibiting PPO activity.

POD plays a crucial role in the oxidative metabolism and browning processes of fresh-cut yam, with its active changes closely linked to the degradation of yam quality. As storage duration increased, POD activity in all treatment groups exhibited an upward trend, suggesting the activation of mechanisms related to POD synthesis (Figure 9D). The most significant increase in POD activity occurred in the 0 MIC group, which reached 7899.96 U/g by day 15 of storage. In contrast, the rise in POD activity in treatment groups supplemented with 1/2 MIC, MIC, and 2 MIC Geraniol was markedly lower than that of the control group (*p* < 0.05). After 15 days, POD activity in the MIC group measured 3780.49 U/g, while in the 2 MIC group, it was 641.97 U/g. These findings suggest that Geraniol can alleviate the adverse effects of oxidative browning on quality by regulating the increase in POD activity in fresh-cut yam.

#### 2.3.4. Effect of Geraniol on the MDA Content of Fresh-Cut Yams

**Figure 10:**
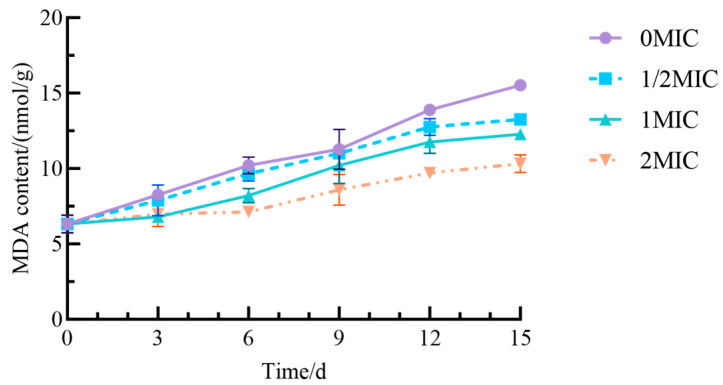
Effect of Geraniol on the MDA content of fresh-cut yam.

MDA is a significant product of lipid peroxidation; thus, elevated MDA levels in plant tissues indicate a greater degree of lipid peroxidation in cell membranes and more severe membrane damage. Variations in MDA content reflect the extent of oxidative damage to fresh-cut yam during storage [42]. The MDA content in all groups of fresh-cut yam increased over time, leading to intensified cell membrane damage (Figure 10). The most pronounced increase in MDA content occurred in the 0 MIC group, which reached 15.3 nmol/g by day 15 of storage. In contrast, groups treated with 1/2 MIC, MIC, and 2 MIC Geraniol exhibited a significantly smaller increase in MDA content compared to the control group (*p* < 0.05). Furthermore, higher concentrations of Geraniol correlated with a slower rate of increase in MDA levels. These results suggest that Geraniol can inhibit lipid peroxidation, reduce MDA production, and thereby lessen oxidative damage in yam.

#### 2.3.5. Effect of Geraniol on the Total Bacterial Count in Fresh-Cut Yams

**Figure 11:**
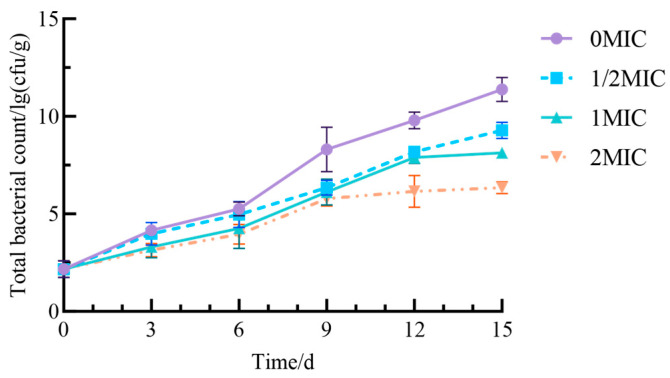
Effect of Geraniol on the total colony count of fresh-cut yam.

As storage time increased, the total plate count of fresh-cut yams across all groups (Figure 11) exhibited an upward trend. This phenomenon can be attributed to the storage environment, which creates favorable conditions for microbial proliferation, while tissue damage from the fresh-cutting process further exacerbates microbial contamination. The most significant increase in total colony count was observed in the 0 MIC group, which reached approximately 11.38 lg (cfu/g) after 15 days. In contrast, the groups treated with 1/2 MIC, MIC, and 2 MIC of Geraniol demonstrated a markedly smaller increase in total colony count compared to the control group (*p* < 0.05). Additionally, as the concentration of Geraniol increased, the total colony count in the fresh-cut yams decreased. These results suggest that Geraniol effectively reduces the total colony count in fresh-cut yams by inhibiting the growth and reproduction of microorganisms, thereby enhancing storage safety.

## 3. Materials and Methods

### 3.1. Antifungal Activity of Geraniol Against P. polonicum

#### 3.1.1. Strains and Culture Conditions

Geraniol (98%, analytical grade) was obtained from Shandong Keyuan Biochemical Co., Ltd. (Laizhou, China). *P. polonicum* (CICC40167) was supplied by the China Industrial Culture Collection Center in Beijing and was cultured at 28 °C.

#### 3.1.2. Determination of *P. polonicum* Colony Expansion

Geraniol concentrations were established at 0, 0.05, 0.1, 0.2, 0.3, 0.4, and 0.5 mL/L. An 8 mm-diameter disk was excised from a *P. polonicum* plate that had been activated for 7 days and positioned at the center of PDA plates containing varying concentrations of the antimicrobial agent. This setup resulted in a culture medium comprising both the antimicrobial agent and the bacterial disk. The Petri dish was then placed in a 28 °C incubator and incubated for 7 days, with daily measurements of colony diameter. The concentration of the antimicrobial agent that inhibited *P. polonicum* growth within 48 h was designated as the minimum inhibitory concentration (MIC), while the concentration that prevented *P. polonicum* growth for 72 h was defined as the minimum fungicidal concentration (MFC) [43] (No colonies grew after the sample was inoculated into fresh medium and subcultured). After 5 days of cultivation on plates, the colony diameter was determined using the cross-counting method, and the inhibition rate of mycelial growth of *P. polonicum* treated with Geraniol was calculated. Following 2 and 5 days of incubation, hyphae from the edges of *P. polonicum* colonies were collected, fixed overnight in 2.5% glutaraldehyde, and centrifuged to remove the supernatant. The samples were washed three times with 100 mM phosphate-buffered saline (PBS), dehydrated using an alcohol gradient, treated with tert-amyl alcohol for 15 min, and subsequently subjected to vacuum freeze-drying for 18 h. After gold-sputtering treatment, the samples were examined under a scanning electron microscope (SEM; 5000× magnification, Guoyi Quantum, Hefei, China) to observe their microscopic features.

#### 3.1.3. Determination of the Relative Electrical Conductivity of *P. polonicum*

Relative electrical conductivity was assessed using a conductivity meter [44]. To begin, 1 mL of *P. polonicum* cultured for 48 h is transferred into a PDB. Following this, the culture is incubated in a shaking incubator at 180 rpm for an additional 48 h, after which it is centrifuged at 10,000 rpm for 10 min. One gram of mycelium is then washed twice with sterile water and resuspended in 100 mL of sterile water. Geraniol is added to achieve final concentrations of 0.3 and 0.6 mL/L. The cultivation continues at 28 °C and 180 rpm. Conductivity measurements are taken from 5 mL of the culture at 0, 2, 4, 6, 8, 10, and 12 h. Subsequently, the culture is boiled for 5 min, and conductivity is measured again. The relative conductivity is calculated using the following formula:

Relative Conductivity (%) = (C_t_ − C_0_)/(C_f_ − C_0_) × 100, (1)

C_t_—Conductivity at a given time point (μs/cm); C_0_—Conductivity at time 0; C_f_—Conductivity measured after boiling for 5 min.

#### 3.1.4. Assessment of Geraniol on the Cell Membranes of *P. polonicum*

Cell membrane integrity was evaluated using the propidium iodide (PI) staining method [45]. Geraniol was incorporated into potato dextrose agar (PDA) at concentrations of 0, 0.0375, 0.075, and 0.15 mL/L. A 20 μL aliquot of *P. polonicum* at a concentration of 10^6^ CFU/mL was inoculated onto the agar, followed by the placement of a sterile coverslip on the PDA plate. The plate was incubated until the mycelium extended to the surface of the coverslip. Subsequently, the coverslip was transferred to a microscope slide, and 10 μL of PI reagent was added to stain the sample for 15 min in the dark. Excess dye was removed by rinsing with sterile water, and the sample was examined under a 20× fluorescence microscope. A 1 mL sample of *P. polonicum* cultured for 48 h was then transferred to potato dextrose broth (PDB) medium and incubated in a shaking incubator at 180 rpm and 28 °C for an additional 48 h. Following incubation, the culture was centrifuged at 10,000 rpm for 10 min to isolate the mycelium. One gram of mycelium was resuspended in 50 mL of 0.9% saline, and Geraniol was added to achieve final concentrations of 0.3 and 0.6 mL/L, with continued incubation at 28 °C and 180 rpm. Supernatant samples were collected at 0, 2, 4, 6, 8, 10, and 12 h. Absorbance was measured at 260 nm (OD260 nm), and protein content was determined using a BCA protein assay kit (Beijing Solabio Technology Co., Ltd., Beijing, China).

#### 3.1.5. Determination of Reactive Oxygen Species Metabolism in *P. polonicum* by Geraniol

Collect *P. polonicum* spores treated with 0.3 and 0.6 mL/L of Geraniol, stain them using DCHF-DA dye, wash them twice with PBS, resuspend in PBS, and observe the staining under a fluorescence microscope. Weigh 0.3 g of mycelium, add 1 mL of PBS buffer pre-chilled to 4 °C, and sonicate at 200 W for 3 s per cycle with 10 s intervals, repeating this process 30 times. Centrifuge the mixture at 8000× *g* at 4 °C for 10 min to obtain the supernatant. MDA content was determined using an MDA detection kit, while the activities of SOD, POD, and CAT were measured with SOD, POD, and CAT detection kits, respectively (Beijing Solabio Technology Co., Ltd., Beijing, China).

#### 3.1.6. Effects of Gernaiol on the Mitochondria of *P. polonicum*

The method utilized involved the extraction of 0.5 g of mycelium to determine ATP content via liquid chromatography [46]. ATPase activity was assessed using an ATPase activity assay kit (Nanjing Jiancheng Biotechnology Research Institute Co., Ltd., Nanjing, China). The mycelium was fixed in 2.5% glutaraldehyde at room temperature for 2 h and subsequently incubated overnight at 4 °C. Following centrifugation, the supernatant was collected, and the sample was washed three times with a 0.1 M PBS solution. After undergoing dehydration, displacement, washing, and drying, the samples were gold-coated for 120 s using an ion-sputtering coating machine and were then observed and photographed with a transmission electron microscope (Hitachi-HT7800, Hitachi High-Technologies Corporation, Tokyo, Japan).

### 3.2. Effects of Geraniol on the Quality of Fresh-Cut Yam Following Infection by P. polonicum

Fresh iron-rod yams were peeled and sliced into 8 mm thick pieces. The mucilage was removed by washing with clean water until the distilled water ran clear. The slices were then immersed in 75% ethanol for 1 min. Using a pipette, 0.1 mL of spore suspension was evenly inoculated onto the surface of each fresh-cut yam sample. The samples were placed in a 9 L preservation box containing solutions with final concentrations of 0, 0.15, 0.3, and 0.6 mL/L of Geraniol (by volume). After 20 h of fumigation, the fresh-cut yams were stored in self-sealing bags at 10 °C for 15 days, with the untreated group serving as the control. Samples were collected on days 0, 3, 6, 9, 12, and 15. Sensory evaluations were conducted by 10 trained assessors aged between 25 and 50. The samples were placed in standardized containers, each labeled with a three-digit random code to ensure the validity of the blind test (Table 2). The weight loss rate (%) of the fresh-cut yams was calculated as (initial weight − actual weight)/initial weight × 100. The L*, a*, and b* values of the fresh-cut yam slices were measured using a CM-5 colorimeter (Shenzhen, China). Yam texture was evaluated through a penetration test (TA-XT plus Texture Analyser, Godalming, UK; P2 probe). Five points are selected daily along the cross-section of the fresh-cut yam, with speeds of 3, 1 and 3 mm/s set for the pre-measurement, mid-measurement and post-measurement phases, respectively. The minimum detectable force is 5 g, and the penetration depth is 3 mm. MDA content was determined using an MDA assay kit. The activities of CAT, SOD, PPO, and POD were measured using the CAT Activity Assay Kit, SOD Activity Assay Kit, PPO Activity Assay Kit, and POD Activity Assay Kit (Beijing Solabio Technology Co., Ltd.). The total plate count of fresh-cut yam was determined following GB 4789.2-2022 [47], which outlines the “Microbiological Examination of Foods—Enumeration of Molds and Yeasts.”

### 3.3. Statistical Analysis

All experiments were performed in triplicate. The results were analyzed with SPSS software (Version 25; IBM Corp., Armonk, NY, USA). Significant differences were identified at the *p* < 0.05 level using one-way analysis of variance (ANOVA) and Bonferroni statistical tests. Figures and charts were created using Prism 10.1.2 software.

## 4. Conclusions

This study elucidates the inhibitory mechanism of Geraniol against *P. polonicum* and its efficacy in preserving fresh-cut Chinese yam. The results indicate that MIC and MFC of Geraniol against *P. polonicum* are 0.3 mL/L and 0.4 mL/L, respectively. The inhibitory mechanism displays multi-target characteristics: it disrupts cell membrane integrity, resulting in the leakage of intracellular nucleic acids and proteins, as well as an increase in relative conductivity; it induces a surge of ROS, promotes lipid peroxidation, elevates MDA content, and concurrently activates antioxidant enzymes such as SOD, POD, and CAT; it also interferes with energy metabolism, significantly reducing ATP content and ATPase activity within the cell, which leads to pronounced mitochondrial vacuolization and cristae rupture.

In the preservation of fresh-cut Chinese yams, Geraniol fumigation treatment significantly delayed the deterioration of sensory quality and weight loss. This treatment effectively maintained hardness and color, as indicated by L*, a*, and b* values, while suppressing the activity of browning-related enzymes, specifically PPO and POD. Additionally, it enhanced the activity of antioxidant enzymes, including CAT and SOD, reduced MDA accumulation, and decreased total colony count. Geraniol inhibits *P. polonicum* through multiple mechanisms, such as disrupting cell membranes, inducing oxidative stress, and interfering with energy metabolism. As a natural food-grade preservative, Geraniol demonstrates promising potential for extending the shelf life of fresh-cut Chinese yams.

## Figures and Tables

**Table 1:** Sensory scores for fresh-cut yam.

Group	Storage Days/d					
	0	3	6	9	12	15
0 MIC	100.00	78.25 ± 3.87 d	62.23 ± 1.85 d	58.23 ± 1.02 d	49.33 ± 3.21 d	45.65 ± 4.22 d
1/2 MIC	100.00	90.27 ± 0.34 c	86.29 ± 1.56 c	84.61 ± 1.69 c	78.69 ± 1.62 c	60.34 ± 4.15 c
MIC	100.00	95.13 ± 1.67 b	93.33 ± 1.11 b	89.11 ± 0.34 b	81.21 ± 0.78 b	70.12 ± 2.15 b
2 MIC	100.00	98.25 ± 0.42 a	96.57 ± 0.76 a	93.21 ± 0.52 a	89.41 ± 1.07 a	80.35 ± 0.27 a

Note: Different lowercase letters following the data indicate a statistically significant difference (*p* < 0.05).

**Table 2:** Sensory evaluation criteria for fresh-cut yam.

Indicator	Standard	Score
Appearance	White in color, with a glossy finish	18–25
	The color is white or pale yellow, and the luster has diminished somewhat	9–17
	Overall dull, with patches of yellowish-brown or dark red	1–8
Smell	Chinese yam has a rich aroma and no unpleasant odor	18–25
	Chinese yam has a mild flavor and no unpleasant odor.	9–17
	No smell of yam, but has an off-odor	1–8
Texture	Hard and brittle in texture, with no signs of softening	18–25
	The texture has softened slightly	9–17
	Severe softening	1–8
Organizational status	The cut surface is clean and free of decayed tissue	18–25
	Some sap has leaked out, and there is slight rot	9–17
	Rotting, with significant loss of sap	1–8

## Data Availability

Data are contained within the article.
